# Self-Reported Utilization of International (ACVIM Consensus) Guidelines and the Latest Clinical Trial Results on the Treatment of Dogs with Various Stages of Myxomatous Mitral Valve Degeneration: A Survey among Veterinary Practitioners

**DOI:** 10.3390/ani14050772

**Published:** 2024-02-29

**Authors:** Marie D. B. van Staveren, Esther Muis, Viktor Szatmári

**Affiliations:** Department of Clinical Sciences, Faculty of Veterinary Medicine, Utrecht University, Yalelaan 108, 3584 CM Utrecht, The Netherlands

**Keywords:** ACE inhibitor, benazepril, enalapril, evidence-based medicine, furosemide, loop diuretics, pimobendan, pulmonary edema, ramipril, spironolactone, torasemide, transcatheter egde-to-edge repair

## Abstract

**Simple Summary:**

The most common heart disease in dogs is a leaky mitral valve. This disease is incurable and slowly progressive. However, with appropriate therapy, prolonged longevity and improved quality of life can be reached. International guidelines are available to help veterinarians treat these dogs according to the latest insights. However, these guidelines might become outdated because scientific evidence can only be reviewed until about a year before their publication. Moreover, disagreements among the panelists who prepare the guidelines, and newer study results challenging the guidelines can make it difficult for practicing veterinarians to choose the right treatment. Our study aimed to investigate, using a survey, how Dutch and Belgian veterinary practitioners treat dogs with a leaky mitral valve. From the 363 responses, we concluded that the more advanced the disease stage is, the more variation there is among the recommended drug regimes. The earliest asymptomatic stage of the disease was treated correctly by 93% of the respondents, whereas the more advanced asymptomatic stage was treated by 67% of the respondents correctly. For the treatment of the symptomatic stage of the disease, 16 various drug combinations were reported, of which only 2 are considered correct, recommended by 48%. No respondents recommended surgery.

**Abstract:**

Background: Myxomatous mitral valve degeneration is the most common canine heart disease. Several clinical trials have investigated various treatments. The latest recommendations are published in the ACVIM consensus guidelines (2019). Our study aimed to investigate how closely veterinary practitioners apply the treatment recommendations of these guidelines and the latest clinical trials. Methods: An online survey was sent to Dutch and Belgian veterinary practices via digital channels. Results: The data from 363 fully completed surveys were analyzed. For stage B1 disease, 93% recommended, correctly, no treatment. For stage B2 disease, 67% of the respondents recommended starting pimobendan as monotherapy. For chronic treatment of stage C disease, 16 different drug combinations were mentioned, but nobody recommended surgery. Only 48% of the respondents recommended the only evidence-based drug combination: a loop diuretic with pimobendan. A concerning finding was the simultaneous prescription of two loop diuretics, by 19% of the respondents. Conclusions: Treatment recommendations showed an increasing variation with more advanced disease stages from B1 through B2 to C. This reflects the increasing disagreement among the panelists who prepared the ACVIM consensus guidelines. Practitioners of our study seem to practice more evidence-based medicine than veterinary cardiologists, as it was reported in a recent survey-based study.

## 1. Introduction

Myxomatous mitral valve degeneration (MMVD) is the most common heart disease in dogs [1,2,3,4,5,6,7,8]. This disease is incurable, as the etiology and exact pathogenesis are still unknown [1,4,7,8]. Mitral regurgitation progressively worsens over the years, causing a left apical systolic murmur on auscultation with increasing intensity, along with chronic left ventricular volume overload [1,2,3,4,5]. Despite progressive and ongoing cardiac remodeling, affected dogs tend to remain free of clinical signs for years and often for life [1,2,4,6]. The appearance of clinical signs, typically as a result of cardiogenic pulmonary edema, i.e., congestive left-sided heart failure (CHF), indicates a very advanced disease stage, which manifests as exercise intolerance, tachypnoea, and dyspnea [1,2,3,4,5,7]. According to the published guidelines of the American College of Veterinary Internal Medicine (ACVIM, 2019), the subclinical stage of the disease is divided into an early stage, when the left atrium and left ventricle are of normal size (stage B1), and an advanced stage, when left ventricular eccentric hypertrophy and left atrial dilatation are present (stage B2) [4]. Because MMVD is an incurable disease, the treatment goals in the subclinical stage are to delay the onset of CHF and to prolong longevity [1,4,6,8,9,10,11,12]. The clinical stage of the disease, caused by CHF, is divided into an early (stage C) and a therapy-resistant late stage (stage D) [4]. The primary goal of treatment in stages C and D is to improve the quality of life by resolving the clinical signs [4,5,7,13,14,15,16,17,18,19,20,21,22,23,24].

In addition to defining the various disease stages, the ACVIM consensus guidelines also give treatment recommendations for each stage [4]. Because even the panelists failed to reach a consensus for several stages, we suspect that practicing veterinarians struggle with applying the published treatment recommendations in their practice. Selecting the correct treatment might have become even more complex because several milestone clinical trials were published after the publication of the latest ACVIM guidelines of 2019 [10,18,19].

The goal of our survey-based study was to reveal whether veterinary practitioners utilize the treatment recommendations of the latest ACVIM consensus guidelines [4] or apply the results of the latest clinical trials for those stages of MMVD for which evidence-based data are available, i.e., stages B1, B2, and C [8,10,11,12,18,19].

## 2. Materials and Methods

An online questionnaire on therapy for MMVD was sent out in the Dutch language to Belgian and Dutch veterinary practices as part of a larger survey. The web-based questionnaire (powered by Qualtrics, Provo, UT, USA) was distributed via various digital channels between 1 July 2021 and 15 August 2021. Participation was anonymous and voluntary. Taking part in the survey was encouraged by a lottery to receive free entrance tickets to a Dutch veterinary cardiology symposium (Hart voor de Praktijk). The veterinarians needed to provide informed consent on the initial page of the questionnaire before they could start the survey. The link to the survey was shared with individual veterinarians and veterinary practices via multiple sources: the electronic newsletter of the Royal Dutch Veterinary Association (KNMvD), a Dutch veterinary online discussion group (Het Dierenartsen Gilde, hosted by Facebook), the mailing list of an agency that specializes in organizing veterinary events (Vitaux), the newsletter of a global veterinary care provider chain (Evidensia), and the newsletters of two pharmaceutical companies (Boehringer Ingelheim and Vetoquinol).

The survey consisted of 18 questions, which were either multiple-choice questions with an additional open-answer option or short-answer open questions. The survey comprised questions about the characteristics of the responding veterinarians, such as country of education, country of practice, years of experience as a practicing veterinarian, and size and type of practice, the latter referring to the percentage of companion animals. The content-related questions focused on the therapeutic approach for stages B1, B2, and C of MMVD. The questionnaire was presented in a set order, and it was required to answer each question in order to proceed to the next one. Before submitting, the respondents could modify their answers. The questionnaire was part of a larger survey, which consisted of additional questions on diagnosing the various stages of MMVD. The results of the questions on diagnostics are reported in a different paper [25].

The received questionnaires were downloaded from the used digital platform (Qualtrics Provo, UT, USA) and imported into a statistical program (Excel, Microsoft v16.78.3, Redmond, WA, USA). All open answers were sorted into categories before importing the data into another statistical program (SPSS Statistics v28, IBM, Chicago, IL, USA) for statistical analysis. The data are presented as percentages. Correlations among variables were analyzed using Spearman’s correlation coefficient (r). The strength of correlation was classified as follows: 0.91–1.00 (very strong), 0.71–0.90 (strong), 0.51–0.70 (moderate), 0.31–0.50 (weak), and 0.01–0.30 (very weak). *p*-values less than 0.05 were considered statistically significant.

## 3. Results

Out of the 524 questionnaires received, 161 were not included in the analysis because of incompleteness. Consequently, 363 questionnaires underwent analysis.

### 3.1. Characteristics of Respondents

The vast majority of the respondents worked in the Netherlands (97%) and only a small fraction in Belgium (3%). Most of the respondents had their veterinary education in the Netherlands (85%); this was followed by 14% in Belgium. Germany, South Africa, and Poland were the other countries where respondents had their veterinary education (a total of 1%). The respondents’ experience working as veterinary practitioners was grouped into three categories: <5 years (28%), 5–15 years (35%), and >15 years (37%).

**Figure 1:**
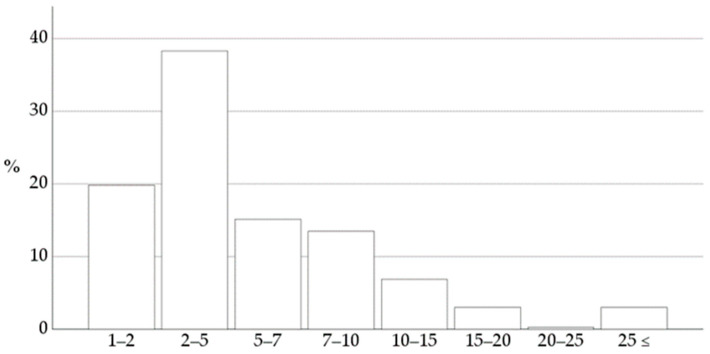
Sizes of the veterinary practices based on the number of veterinarians employed. Most respondents (38%) worked in a practice with 2 to 5 veterinarians [25]. Reprinted with permission.

Regarding the profile of the practice, 18% of the respondents worked in a mixed practice where both small and large animals were welcome, and 82% of the respondents worked in a practice accepting companion animals only [25]. The sizes of the practices where the respondents worked are presented in Figure 1 [25]. The number of responding veterinarians equals roughly 12% of the total practicing veterinarians focusing on companion animals in the Netherlands [26].

### 3.2. Utilizing the ACVIM Consensus Guidelines for Staging of Myxomatous Mitral Valve Degeneration

Of the 363 respondents, 60% reported using the ACVIM guidelines for staging MMVD, and the remaining 40% did not [25].

### 3.3. Pharmacological Recommendation for Dogs with Myxomatous Mitral Valve Degeneration

#### 3.3.1. Pimobendan

The veterinarians were asked based on which diagnostic test(s) and which diagnostic test results they decide to prescribe pimobendan for asymptomatic adult dogs with a heart murmur. The respondents had the option to select multiple answers and provide additional free text. Among the respondents, 96% would prescribe pimobendan and 4% would never prescribe pimobendan for asymptomatic adult dogs with a heart murmur. The respondents based their decision to prescribe pimobendan on the results of echocardiography (80%), thoracic radiography (59%), clinical examination (20%), or they follow the echocardiographer’s recommendation (4%). A total of 13% indicated they prescribed pimobendan to a dog when a pulmonary edema is found, and 2% in cases of pleural effusion. When the respondents were asked to specify the echocardiographic findings that prompt them to prescribe pimobendan, the most common response was that this was recommended by the echocardiographer (41%). The most frequently mentioned echocardiographic criterium for prescribing pimobendan was an enlarged left atrium (35%), and the second most frequent criterium was an enlarged left ventricle (15%). The remaining 12% answered that they prescribe pimobendan when a dog meets the criteria for stage B2 mitral valve disease. Of the respondents who use thoracic radiographs, 67% mentioned an enlarged vertebral heart scale (VHS) [27], 28%—cardiomegaly, 14%—enlarged left atrium, 13%—pulmonary oedema, 3%—an abnormal heart shadow, 2%—dilatation of the lung vessels, 2%—pleural effusion, and 2%—a dorsal shift of the trachea as the criterion to prescribe pimobendan. Only 1% of the respondents mentioned an enlarged vertebral left atrial size (VLAS) [4,28].

The respondents who utilize the ACVIM staging guidelines for MMVD were more likely to prescribe pimobendan for an asymptomatic adult dog with a heart murmur after performing diagnostic imaging tests than respondents who do not utilize the ACVIM guidelines (61% vs. 39%, r = −0.127, *p* = 0.015). The respondents using the ACVIM staging guidelines were more likely to prescribe pimobendan for asymptomatic adult dogs with a heart murmur after diagnosing an enlarged left atrium using echocardiography than respondents who do not use the staging guidelines (37% vs. 14%, *p* ≤ 0.001, r = 0.256). The respondents who utilize the ACVIM staging guidelines are less likely to prescribe pimobendan for asymptomatic adult dogs with a heart murmur based on the echocardiographer’s advice than respondents who do not use the staging guidelines (42% vs. 26%, r = −0.161, *p* = 0.002).

The respondents using the ACVIM staging guidelines were more likely to prescribe pimobendan for asymptomatic adult dogs with a heart murmur based on an enlarged VHS than respondents not using the guidelines (45% vs. 27%, r = 0.175, *p* ≤ 0.001). The respondents using the ACVIM staging guidelines were less likely to prescribe pimobendan for asymptomatic adult dogs with a heart murmur utilizing physical examination findings than the respondents who do not use the guidelines (27% vs. 14%, r = −0.162, *p* = 0.002).

More experienced respondents (≥15 years) were more likely to decide to prescribe pimobendan utilizing physical examination findings for asymptomatic adult dogs with a heart murmur than respondents with less experience (≤5 years) (25% vs. 14%, r = 0.111, *p* = 0.038). Less experienced respondents (≤5 years) prescribe pimobendan for asymptomatic adult dogs with a heart murmur with an enlarged left atrium on echocardiography more often than more experienced respondents (≥15 years) (33% vs. 21%, r = −0.114, *p* = 0.033).

The respondents working in large practices (≥25 veterinarians) were more likely to prescribe pimobendan after diagnosing an enlarged left atrium using echocardiography for asymptomatic adult dogs with a heart murmur than respondents who work in small (1 to 2 veterinarians) practices (40% vs. 14%, r = 0.154, *p* ≤ 0.003).

#### 3.3.2. Angiotensin-Converting Enzyme Inhibitors

The veterinarians were asked based on which diagnostic test(s) and which diagnostic test results they decide to prescribe an angiotensin-converting enzyme inhibitor (ACE inhibitor) for asymptomatic adult dogs with a heart murmur. The respondents had the option to select multiple answer options and provide additional free text. Among the respondents, 76% would never prescribe an ACE inhibitor, but 34% would do so for asymptomatic adult dogs with a heart murmur. The respondents base their decisions on the results of echocardiography (20%), thoracic radiography (7%), or physical examination (6%). Only 4% prescribe an ACE inhibitor based on the echocardiographer’s advice.

The respondents using the ACVIM staging guidelines are less likely to prescribe an ACE inhibitor for asymptomatic adult dogs with a heart murmur compared to those who do not utilize the guidelines (18% vs. 32%, r = −0.165, *p* = 0.002).

Experienced (≥15 years) and less experienced (≤5 years) respondents were equally unlikely to prescribe an ACE inhibitor for asymptomatic adult dogs with a murmur (74% vs. 79%, r = −0.036, *p* = 0.5).

The respondents working in large practices (≥25 veterinarians) were less likely to prescribe an ACE inhibitor for asymptomatic adult dogs with a heart murmur than the respondents who work in small (1 to 2 veterinarians) practices (80% vs. 64%, r = 0.105, *p* = 0.046).

### 3.4. Treatment Recommendations for Dogs with Stage B1 Degenerative Mitral Valve Disease

Among the respondents, 93% would not recommend any treatment for dogs with confirmed stage B1 MMVD (Table 1). However, 7% would recommend starting a life-long daily oral medication. Of this 7%, 93% would recommend pimobendan and 15% would recommend a loop diuretic. None of the respondents recommended an ACE inhibitor for this disease stage. The respondents using the ACVIM staging guidelines for MMVD are less likely to recommend starting a life-long daily oral medication than those who do not use the guidelines (5% vs. 12%, r = −0.121, *p* = 0.021).

### 3.5. Treatment Recommendations for Dogs with Stage B2 Degenerative Mitral Valve Disease

Among the respondents, 84% would prescribe life-long daily oral medication for dogs with confirmed stage B2 MMVD, and the remaining 16% would not recommend any treatment (Table 2). The ones who would recommend medical treatment reported the following drugs: pimobendan (98%), loop diuretics (14%), ACE inhibitor (4%), and spironolactone (1%). Among those who would prescribe pimobendan, 81% use it as a monotherapy, 13% combine it with a loop diuretic, 2% with an ACE inhibitor, 1% with spironolactone and a loop diuretic, and another 1% with an ACE inhibitor and a loop diuretic. Only 1% of the respondents would prescribe an ACE inhibitor as monotherapy, and another 1% a loop diuretic as monotherapy for dogs with stage B2 MMVD. In total, 67% of the respondents would prescribe pimobendan as monotherapy for a dog with confirmed stage B2 MMVD.

The respondents using the ACVIM staging guidelines would prescribe pimobendan more frequently for dogs with stage B2 MMVD than those who do not utilize the guidelines (89% vs. 74%, r = 0.195, *p* ≤ 0.001). Use of the ACVIM staging guidelines did not influence whether the respondents would prescribe a loop diuretic (11% vs. 12%, r = −0.009, *p*= 0.864) or an ACE inhibitor (3% vs. 4%, r = 0.037, *p* = 0.48).

Experienced (≥15 years) and less experienced (≤5 years) respondents prescribe all three types of drugs with similar frequency—pimobendan (79% vs. 86%, r = 0.338, *p* = 0.345), an ACE inhibitor (4% vs. 2%, r = 0.530, *p* = 0.505), and a loop diuretic (14% vs. 7%, r = 0.077, *p* = 0.148)—for dogs with stage B2 MMVD.

The respondents working in large practices (≥25 veterinarians) and small practices (1 to 2 veterinarians) prescribe pimobendan with equal frequency (81% vs. 80%, r = 0.012, *p* = 0.816). However, ACE inhibitors (0% vs. 6%, r = −0.066, *p* = 0.212) and loop diuretics (0% vs. 14%, r = −0.056, *p* = 0.286) were prescribed less frequently in large practices, but this difference was not statistically significant.

### 3.6. Treatment Recommendations for Dogs with Stage C Degenerative Mitral Valve Disease

The veterinarians were asked which medications they would prescribe for a dog with stage C disease if they wanted to provide an optimal maintenance therapy, and the owner had no financial concerns. All respondents (100%) would advise a loop diuretic, 78% pimobendan, 17% an ACE inhibitor, and 11% spironolactone. Of the respondents who recommended a loop diuretic, 82% would prescribe furosemide and 36% torasemide (Table 3). Other recommendations included the following: 2% of the respondents recommend immediate euthanasia, 0.3% recommended digoxin, and 0.3% would not start treating a dog with stage C disease but would recommend immediate referral. None of the respondents mentioned surgery as a treatment option. Medical treatment recommendations consisted of 16 drug combinations: 35% recommended pimobendan with furosemide, 13%—pimobendan with torasemide, 9%—pimobendan with furosemide and torasemide, 9%—an ACE inhibitor with pimobendan and furosemide, 4%—a combination of furosemide with torasemide, 3%—an ACE inhibitor with pimobendan, spironolactone, and furosemide, 3%—pimobendan with spironolactone and furosemide, 2%—an ACE inhibitor with furosemide, 2%—an ACE inhibitor with pimobendan, spironolactone, furosemide, and torasemide, 2%—pimobendan with spironolactone, furosemide, and torasemide, 1%—pimobendan with spironolactone and torasemide, 1%—spironolactone with furosemide and torasemide, 1%—an ACE inhibitor with pimobendan, spironolactone, and torasemide, 1%—an ACE inhibitor with pimobendan, furosemide, and torasemide, 1%—an ACE inhibitor with pimobendan and torasemide, and 0.3%—an ACE inhibitor combined with torasemide. A combination of two loop diuretics simultaneously, i.e., furosemide with torasemide, was recommended by 19% of the respondents.

No correlation was found between prescribing pimobendan and using the ACVIM staging guidelines (80% vs. 75%, r = −0.06, *p* = 0.257), the size of the practice the respondent worked for (72%—small with 1–2 veterinarians vs. 80%—large practices with more than 25 veterinarians, r = 0.061, *p* = 0.249), or the years of experience (76%—less than 5 years vs. 75%—more than 15 years of experience, r = −0.015, *p* = 0.775).

No correlation was found between prescribing an ACE inhibitor and using the ACVIM staging guidelines (17% vs. 19%, r = −0.025, *p* = 0.640), the size of practice (22%—small vs. 20%—large practice, r = −0.012, *p* = 0.827), or the years of experience (19%—less than 5 years vs. 12%—more than 15 years, r = −0.084, *p* = 0.117).

No correlation was found between prescribing spironolactone and using the ACVIM staging guidelines (14% vs. 7%, r = 0.109, *p* = 0.038), the size of practice (14%—small vs. 20%—large practice, r = −0.056, *p* = 0.289), or the years of experience (15%—less than 5 years vs. 9%—more than 15 years, r = −0.074, *p* = 0.168).

The respondents using the ACVIM staging guidelines would prescribe furosemide with torasemide more frequently for dogs with stage C MMVD than those who do not utilize the guidelines (22% vs. 12%, r = 0.124, *p* = 0.018). The respondents working in large practices (≥25 veterinarians) were more likely to prescribe furosemide with torasemide than the respondents who work in small (1 to 2 veterinarians) practices (18% vs. 13%, r = 0.118, *p* = 0.024). No correlation was found between the years of experience and prescribing furosemide with torasemide (21%—less than 5 years vs. 15%—more than 15 years, r = −0.06, *p* = 0.253).

## 4. Discussion

The most striking finding of the present study is that the respondents’ treatment recommendations showed increasing variation for the more advanced disease stages, and consequently, more divergence from evidence-based therapy. Stage B1 MMVD was treated correctly by 93% of the respondents, i.e., no treatment. Stage B2 disease was treated correctly, i.e., monotherapy with pimobendan, by 67% of the respondents. Stage C disease was treated by only 48% of the respondents according to current evidence-based veterinary medicine, i.e., with pimobendan and furosemide (35%) or pimobendan and torasemide (13%).

When the participants were asked about their reasons for prescribing pimobendan for an asymptomatic adult dog with a murmur, a number of surprising responses were found. A total of 13% of the respondents mentioned the presence of pulmonary edema and 2% mentioned pleural effusion. These responses are unexpected, since pulmonary edema would be very unlikely to be present in a dog without any clinical signs, such as exercise intolerance, dyspnea, and tachypnea [1,2,3,4,5,6,7,8,9,29,30,31,32,33,34,35]. Pleural effusion is a very unlikely manifestation of MMVD in dogs [6]. Of the respondents who would prescribe pimobendan for an asymptomatic adult dog with a heart murmur, 59% would do this based on thoracic radiographic findings. Of these respondents, 67% use the VHS and only 1% uses VLAS to establish the indication. This large difference might be caused by the fact that the VHS was introduced almost three decades ago, and it is, therefore, probably better known to veterinarians than the more recently described radiographic assessment methods for left atrium size, such as the VLAS [27,28,36,37]. However, the use of the VLAS is recommended in the ACVIM guidelines [4]. We also found that respondents who use the ACVIM staging guidelines more likely prescribe pimobendan based on the results of diagnostic imaging findings, echocardiography, and thoracic radiography. Because the respondents tend to follow the recommendations of the veterinarian who perform the echocardiography, it is of utmost importance that this veterinarian is trained not only for performing an echocardiographic study, but has also an up-to-date knowledge on treatment too. For this reason dogs with a murmur are ideally referred to a certified veterinary cardiologist. Our study shows that less experienced respondents (≤5 years) prescribe pimobendan for asymptomatic dogs with a heart murmur with an enlarged left atrium on echocardiography more often than more experienced respondents (≥15 years). Despite the weak correlation, this observation stands out, suggesting that veterinarians who have graduated more recently might have acquired greater skills in basic echocardiography. In contrast, the experienced respondents (≥15 years) use physical examination more often than the less experienced respondents (≤5 years) when justifying their choice for initiating pimobendan. Despite a weak correlation, this result suggests that experienced veterinarians might have greater confidence in their clinical assessments, like grading murmur intensity [38].

Before the publication of the EPIC study [8], veterinarians tended to treat asymptomatic dogs with preclinical stages of MMVD with ACE inhibitors, which was also recommended in the first edition of the ACVIM consensus guidelines [24]. A recent study conducted in the United Kingdom showed a significant negative trend toward the prescription of ACE inhibitors for dogs with MMVD [39]. Although administering an ACE inhibitor for stage B disease might be a theoretically logical choice, no clinical trials have proved their efficacy in postponing the onset of CHF so far [11,12]. We conclude that the majority of veterinarians who participated in our survey practice evidence-based veterinary medicine, as 76% would never prescribe an ACE inhibitor for dogs with stage B MMVD. Interestingly, these veterinarians are more likely to use the ACVIM staging guidelines (as ACE inhibitors are recommended by half of the panelists) than those who do not utilize the guidelines [25]. In addition, we found that veterinarians working in large practices (≥25 veterinarians) are significantly less likely to prescribe ACE inhibitors for asymptomatic adult dogs with a heart murmur than the respondents who work in small practices. This suggests that small veterinary practices might practice less evidence-based than large ones. This finding is not surprising as larger practices often offer the possibility for individual veterinarians to focus on certain organ systems, such as cardiology, in contrast to small practices, where veterinarians must deal with all aspects of veterinary medicine. Moreover, larger practices are more likely to have developed protocols for common diseases, have patient rounds, and organize continuing education sessions for themselves.

For stage B1 disease, the ACVIM guidelines do not recommend any treatment [4]. In line with the guidelines, currently, there is no evidence-based medical or dietary treatment that can slow disease progression or prolong longevity in stage B1 MMVD [11,40,41,42]. Despite this uniform recommendation, 7% of our respondents would still start a life-long daily oral medication for dogs in stage B1, of which 93% would prescribe pimobendan. This practice is ill-advised as the efficacy of pimobendan in this stage has not been investigated. Moreover, a small study showed that chronic pimobendan administration resulted in a worsening of the degenerative lesions of the mitral valve in twelve beagle dogs with naturally occurring stage B1 MMVD [43]. Another argument against starting medical therapy in this stage is that around two-thirds of dogs with stage B1 MMVD would never progress to B2 [1,4]. Therefore, pimobendan would not have any benefit and it might even potentially be harmful, while the pet owner spends money unnecessarily for years. According to the results of a recently published study, which investigated how veterinary cardiologists treated dogs with MMVD in the period between 2015 and 2018, 15% would recommend a medical therapy for dogs in stage B1, of which 69% prescribed an ACE inhibitor and 41% prescribed pimobendan [44]. In this regard, the respondents of our survey, who were practitioners, practice more evidence-based medicine than board-certified veterinary cardiologists.

For stage B2 disease, the ACVIM guidelines recommend pimobendan treatment based on the results of the EPIC study [4,8]. Only two-thirds of the respondents of our study were aware of the benefit of starting pimobendan as a monotherapy in stage B2 of MMVD. This rate is similar to the percentage of veterinary cardiologists (71%) who would recommend pimobendan for dogs in stage B2 MMVD after the publication of the EPIC study [44]. For stage B2, 19% of the respondents of our survey would add other medications, such as an ACE inhibitor or a loop diuretic. Although clinical trials are lacking regarding the use of loop diuretics for the subclinical stage of MMVD, these medications are indicated only for the treatment of congestive heart failure, i.e., stage C [4,24]. Starting diuretic treatment in stage B2 cannot prevent or delay the occurrence of CHF, but it might lead to dehydration, hypokalemia, diuretic resistance, activation of the renin-angiotensin-aldosterone system, and last but not least to incontinent pets. There are no studies that have ever documented a positive effect of an ACE inhibitor for stage B2 MMVD [11,12,45,46]. If any scientific evidence had been available for the positive effect of ACE inhibitors in stage B2 MMVD at the time when the EPIC study, the largest and probably most carefully designed clinical trial in veterinary cardiology, was designed and conducted, it should have been used in the non-pimobendan arm of the study instead of placebo because of ethical reasons [8]. Interestingly, half of the panelists who participated in writing the latest ACVIM consensus guidelines recommend an ACE inhibitor for dogs with stage B2 MMVD despite a lack of scientific evidence, as it was shown in the SVEP trial (published in 2002) and the Vetproof study (published in 2007) [11,12]. Even more surprising is that a recently published study showed that 64% of veterinary cardiologists prescribe an ACE inhibitor for dogs with stage B2 MMVD [44]. In contrast, only 4% of the respondents of our study would recommend an ACE inhibitor for dogs with stage B2 MMVD. This is a remarkable difference, especially if we consider that the participants of our study were first-opinion veterinary practitioners. We conclude that also for treating dogs with stage B2 MMVD, the general veterinary practitioners of our survey seem to practice more evidence-based medicine than board-certified veterinary cardiologists in the countries where the survey was conducted [44]. However, we must admit that one-third of the respondents of our study would consider prescribing an ACE inhibitor for asymptomatic adult dogs with a heart murmur.

For stage C disease, i.e., CHF or cardiogenic pulmonary edema, a loop diuretic, such as furosemide, is the only essential life-saving drug, even in the absence of clinical trials [4,24]. Based on theoretical considerations and extrapolation from human cardiology, several other drugs have been investigated in addition to loop diuretics for chronic maintenance therapy of dogs with stage C MMVD. Since the publication of the QUEST study in 2008, pimobendan has become a routine addition to the diuretic treatment [14]. Because chronic furosemide treatment activates the renin–angiotensin–aldosterone system, theoretical benefits from adding an ACE inhibitor have long been recommended [4,24,47,48]. Both the first and second editions of the ACVIM guidelines on MMVD recommend adding an ACE inhibitor to furosemide and pimobendan, i.e., the so-called triple therapy, for the chronic treatment of dogs with stage C MMVD [4,24]. The guidelines base their advice on the IMPROVE study, which was conducted on the hemodynamic, clinical, and echocardiographic effects of adding enalapril to the conventional therapy for heart failure, furosemide alone at that time, in 22 dogs with naturally acquired heart failure due to MMVD [16]. However, this study monitored these dogs for 21 days only, and did not investigate survival [16]. Although a placebo-controlled, double-blind study, the BENCH study, which investigated the addition of benazepril to furosemide therapy in dogs with stage C MMVD, did show a survival benefit, this study was conducted before pimobendan became available for dogs [15]. However, the very first clinical trial, the VALVE study, which compared double with triple therapy, i.e., adding an ACE inhibitor, ramipril, to pimobendan and furosemide treatment, did not show any beneficial effect on survival in dogs with CHF secondary to MMVD [18]. The VALVE trial was published in 2020, after the latest version of the ACVIM consensus guidelines [4].

For dogs with stage C MMVD, the latest ACVIM guidelines recommend even the addition of spironolactone to the triple therapy [4]. This recommendation is based on theoretical framework of inhibiting the renin–angiotensin–aldosterone system at more than one point to prevent aldosterone breakthrough [48], and the results of an unreliable study on dogs [13,49]. The controversies of the mentioned study mainly arise from methodological imprecisions and incongruences to identify dogs with CHF [13,49]. Therefore, one cannot state that the dogs included in that study were dogs with stage C MMVD according to the current ACVIM classification system [4]. Currently, there are no published studies where the ACVIM staging system was used and the potential benefit of adding spironolactone exclusively to the standard therapy of pimobendan and a loop diuretic was investigated in dogs with stage C MMVD. In a study regarding dogs with naturally occurring MMVD, aldosterone breakthrough was present in about 30% of the dogs with stage C disease [50]. A more recent study, the BESST study, which was published in 2021, after the appearance of the latest ACVIM consensus guidelines [4], showed that the combination of benazepril and spironolactone with furosemide was more effective in improving clinical signs and reducing the risk of cardiac death in dogs with stage C MMVD than benazepril with furosemide alone [19]. Though the difference was statistically significant, the roughly 1 month difference is not considered clinically meaningful. In addition, the dogs in the BESST study were not treated with pimobendan, which would currently be an extremely unusual and even unethical scenario. Therefore, it remains unknown if adding an ACE inhibitor and spironolactone to pimobendan and furosemide therapy is of any benefit to dogs with stage C MMVD.

Furosemide is the first-choice loop diuretic advised by the most recent ACVIM guidelines for the treatment of dogs with CHF secondary to MMVD [4]. Torasemide is only recommended for dogs in which controlling congestive signs during hospitalization with furosemide is difficult or has only limited success [4]. For the treatment of CHF, several studies have shown that torasemide, a more potent loop diuretic than furosemide, is not inferior to furosemide for dogs with MMVD [20,51]. Moreover, there are some additional theoretical benefits, such as a longer duration of action and a higher bioavailability compared to furosemide [20,51]. In our study, a third of the respondents would prescribe torasemide for dogs with CHF. Surprisingly, the combination of two loop diuretics, i.e., furosemide and torasemide, was recommended by a fifth of the respondents of our study. This was an unexpected and concerning finding. This combination is strongly discouraged because of their additive and unpredictable diuretic effects, which can lead to azotemia and even to acute renal failure [52]. Where the idea of combining furosemide with torasemide comes from is unclear, but respondents might use a frequently consulted veterinary website where this combination is mentioned [53]. Even more surprising is that the respondents who utilize the ACVIM guidelines, as well as the respondents who work in large practices, prescribe furosemide with torasemide significantly more often than the respondents who do not use the ACVIM staging guidelines or work in a small practice.

Based on the currently available scientific evidence, we consider the combination of pimobendan with a loop diuretic, i.e., either furosemide or torasemide, the correct medical treatment for dogs with stage C MMVD [13,14,18,19,49,54,55]. Close to half (48%) of the respondents of our study would give this combination to a dog with stage C MMVD, which is not the actual recommendation of the ACVIM guidelines [4]. Although we found a weak positive correlation between recommending spironolactone and utilizing the ACVIM staging guidelines, the combination of furosemide, pimobendan, an ACE inhibitor, and spironolactone was only given by 3% of our respondents. In this respect, again, the respondents of our study are also more likely to practice evidence-based veterinary medicine for stage C disease than to follow the recommendations of the currently outdated and in some aspects biased ACVIM guidelines [4,13,14,18,19,49,54,55].

Interestingly, none of the respondents recommended surgical treatment at any of the disease stages, even though open heart surgery and transcatheter edge-to-edge repair techniques are available options for dogs with advanced stages of MMVD in Europe [56,57,58,59,60]. According to the ACVIM guidelines, a successful surgical intervention is the only way to reach a lower disease stage from C to B [4]. A possible reason for not recommending valve surgery is that at the time of the survey, no surgical options were available in the countries where the study was conducted. Another reason could be cultural, in that Dutch veterinarians do not expect Dutch owners to spend large amounts of money on cardiac surgery, especially abroad, in elderly dogs. Similarly, nobody mentioned left atrial decompression as a palliative and minimally invasive interventional procedure using transseptal puncture [23]. The most likely reason for this finding is that the application of this technique in dogs is fairly new, and therefore, it is not widely known nor used. Transcatheter edge-to-edge repair (TEER) is a promising new surgical treatment modality for selected dogs with MMVD [60]. This technique is much less invasive and therefore less risky and more affordable than open heart surgery. However, there are several limiting factors, such as the size of the heart, the valve anatomy and valve pathology, why TEER cannot be performed in each dog with advanced MMVD. Also it is not yet clear in which disease stage TEER would offer the greatest clinical benefit. Long-term follow up information on a large number of dogs is required before evidence-based recommendation can be made for pet owners.

## 5. Limitations

Our study depended on self-reported statements from veterinarians, which might be a source of bias. In addition, the prize to attend a cardiology conference might have selected responders to participate in the survey who were most interested in companion animal cardiology. Therefore, the knowledge of the general veterinarian, i.e., without specific interest in cardiology, could be lower than observed in this study. Despite designing the questions as objectively as possible, there is a possibility that the way they were framed influenced the answers.

Given the fact that only 3% of the responses came from veterinarians practicing in Belgium, the results primarily reflect the practices of Dutch veterinarians. Consequently, a comparison of the prescription habits of veterinarians between these two countries could not be performed. Further limitations are that no information on the degrees and qualifications of the respondents is available (e.g., PhD and certificates), and no questions were included to determine if the respondents knew how to interpret the guidelines correctly, e.g., if they knew the levels of evidence and classes of recommendation, and how to interpret them.

Our survey did not ask questions about stages A and D of MMVD because of an absence of clinical trials in these stages.

## 6. Conclusions

Identifying heart disease that causes a murmur and adequately staging the disease in asymptomatic adult dogs with a heart murmur is essential for selecting the right therapy, and with that, trying to prolong the subclinical stage of the disease.

Our results show an increasing degree of heterogeneity in the treatment recommendations for dogs with more advanced disease stages from B1 through B2 to C. This finding reflects exactly the increasing degree of disagreement among the panelists of the ACVIM guidelines for the more severe disease stages [4].

The majority of respondents of our study were aware of the most up-to-date evidence-based treatment recommendations originating from the most current ACVIM consensus guidelines and milestone clinical trials. However, an unexpected and concerning finding is the surprisingly high percentage of respondents (one-fifth) who prescribe two loop diuretics simultaneously for dogs with stage C disease.

## Figures and Tables

**Table 1:** Comparing the treatment recommendations of the most recent ACVIM guidelines (2019) and the findings of the present survey for dogs with stage B1 chronic mitral valve disease. ACE inhibitor = angiotensin-converting enzyme inhibitor.

Stage B1 MMVD	Survey Respondents	ACVIM Guidelines, 2019
No therapy	93%	100%
Therapy	7%	0%
Pimobendan	93%	
ACE inhibitors	0%	
Loop diuretics	15%	
Spironolactone	0%	

**Table 2:** Comparing the treatment recommendations of the most recent ACVIM guidelines (2019) and the findings of the present survey for dogs with stage B2 chronic mitral valve disease. ACE inhibitor = angiotensin-converting enzyme inhibitor.

Stage B2 MMVD	Survey Respondents	ACVIM Guidelines, 2019
Therapy: Yes	84%	100%
**Pimobendan total**	98%	100%
Pimobendan monotherapy	81%	50% of panelists
**ACE inhibitors total**	4%	50% of panelists
ACE inhibitor monotherapy	1%	0%
**Loop diuretics total**	14%	0%
Loop diuretics monotherapy	1%	0%
**Spironolactone total**	1%	0%
Spironolactone monotherapy	0%	0%
**Combinations**		
Pimobendan + loop diuretic	13%	0%
Pimobendan + ACE inhibitor	2%	50%
Pimobendan + ACE inhibitor + loop diuretic	1%	0%
Pimobendan + loop diuretic + spironolactone	1%	0%

**Table 3:** Comparing the long-term maintenance treatment recommendations of the most recent ACVIM guidelines (2019) and the findings of the present survey for dogs with stage C chronic mitral valve disease. Pimo = pimobendan; furo = furosemide; tora = torasemide; ACE-i = angiotensin-converting enzyme inhibitor; spiro = spironolactone.

Stage C MMVD	Survey Respondents	ACVIM Guidelines, 2019
Therapy: Yes	100%	100%
Pimobendan (pimo) total	78%	100%
ACE inhibitors (ACE-i) total	17%	100%
Loop diuretics total	100%	100%
Furosemide (furo)	82%	100%
Torasemide (tora)	36%	Some cases instead of furosemide
Spironolactone (spiro) total	11%	Adjunct therapy
**Combinations:**		
Pimo + furo	35%	0%
Pimo + tora	13%	0%
Pimo + furo + tora	9%	0%
Pimo + ACE-i + furo	9%	100%
Furo + tora	4%	0%
Pimo + ACE-i + furo + spiro	3%	Adjunct therapy
Pimo + furo + spiro	3%	0%
Pimo + ACE-i + furo + spiro + tora	2%	0%
Pimo + furo + spiro + tora	2%	0%
ACE-i + furo	2%	0%
Pimo + ACE-i + furo + tora	1%	0%
Pimo + ACE-i + spiro + tora	1%	Some cases
Pimo + ACE-i + tora	1%	Some cases
Pimo + spiro + tora	1%	0%
Furo + spiro + tora	1%	0%
ACE-i + tora	0.3%	0%
**Other:**		For atrial fibrillation
Euthanasia	2%	
Digoxin	0.3%	
Immediate referral	0.3%	

## Data Availability

The data presented in this study are available upon reasonable request to the corresponding author. The data are not publicly available due to privacy reasons.

## Abbreviations

ACE inhibitor = angiotensin-converting enzyme inhibitor; ACVIM = American College of Veterinary Internal Medicine; CHF = congestive heart failure; MMVD = myxomatous mitral valve degeneration; TEER = transcatheter edge-to-edge repair; VHS = vertebral heart scale; VLAS = vertebral left atrial size.

## References

[1] Borgarelli M., Buchanan J.W. (2012). Historical review, epidemiology and natural history of degenerative mitral valve disease. J. Vet. Cardiol..

[2] Mattin M.J., Boswood A., Church D.B., Brodbelt D.C. (2018). Prognostic factors in dogs with presumed degenerative mitral valve disease attending primary-care veterinary practices in the United Kingdom. J. Vet. Intern. Med..

[3] Ljungvall I., Rishniw M., Porciello F., Ferasin L., Ohad D.G. (2014). Murmur intensity in small-breed dogs with myxomatous mitral valve disease reflects disease severity. J. Small Anim. Pract..

[4] Keene B.W., Atkins C.E., Bonagura J.D., Fox P.R., Häggström J., Fuentes V.L., Oyama M.A., Rush J.E., Stepien R., Uechi M. (2019). ACVIM consensus guidelines for the diagnosis and treatment of myxomatous mitral valve disease in dogs. J. Vet. Intern. Med..

[5] Franchini A., Abbott J.A., Tyrrell W., Rosenthal S., Lahmers S., Menciotti G., Crosara S., Häggström J., Borgarelli M. (2021). Predictors of reoccurrence of congestive signs within 180 days after successful treatment of the first episode of congestive heart failure in dogs with myxomatous mitral valve disease. J. Vet. Cardiol..

[6] Gordon S.G., Saunders A.B., Wesselowski S.R. (2022). Asymptomatic canine degenerative valve disease: Diagnosis and current and future therapies. Vet. Clin. N. Am. Small Anim. Pract..

[7] Borgarelli M., Savarino P., Crosara S., Santilli R.A., Chiavegato D., Poggi M., Bellino C., La Rosa G., Zanatta R., Haggstrom J. (2008). Survival characteristics and prognostic variables of dogs with mitral regurgitation attributable to myxomatous valve disease. J. Vet. Intern. Med..

[8] Boswood A., Häggström J., Gordon S.G., Wess G., Stepien R.L., Oyama M.A., Keene B.W., Bonagura J., MacDonald K.A., Patteson M. (2016). Effect of pimobendan in dogs with preclinical myxomatous mitral valve disease and cardiomegaly: The EPIC study—A randomized clinical trial. J. Vet. Intern. Med..

[9] Boswood A., Gordon S.G., Häggström J., Wess G., Stepien R.L., Oyama M.A., Keene B.W., Bonagura J., MacDonald K.A., Patteson M. (2018). Longitudinal analysis of quality of life, clinical, radiographic, echocardiographic, and laboratory variables in dogs with preclinical myxomatous mitral valve disease receiving pimobendan or placebo: The EPIC study. J. Vet. Intern. Med..

[10] Borgarelli M., Ferasin L., Lamb K., Bussadori C., Chiavegato D., D’Agnolo G., Migliorini F., Poggi M., Santilli R.A., Guillot E. (2020). DELay of appearance of symptoms of canine degenerative mitral valve disease treated with spironolactone and benazepril: The DELAY study. J. Vet. Cardiol..

[11] Kvart C., Häggström J., Pedersen H.D., Hansson K., Eriksson A., Järvinen A.K., Tidholm A., Bsenko K., Ahlgren E., Ilves M. (2002). Efficacy of enalapril for prevention of congestive heart failure in dogs with myxomatous valve disease and asymptomatic mitral regurgitation. J. Vet. Intern. Med..

[12] Atkins C.E., Keene B.W., Brown W.A., Coats J.R., Crawford M., DeFrancesco T.C., Edwards N.J., Fox P.R., Lehmkuhl L.B., Luethy M.W. (2007). Results of the veterinary enalapril trial to prove reduction in onset of heart failure in dogs chronically treated with enalapril alone for compensated, naturally occurring mitral valve insufficiency. J. Am. Vet. Med. Assoc..

[13] Bernay F., Bland J.M., Häggström J., Baduel L., Combes B., Lopez A., Kaltsatos V. (2010). Efficacy of spironolactone on survival in dogs with naturally occurring mitral regurgitation caused by myxomatous mitral valve disease. J. Vet. Intern. Med..

[14] Häggström J., Boswood A., O’grady M., Jöns O.J., Smith S., Swift S., Borgarelli M., Gavaghan B., Kresken J.-G., Patteson M. (2008). Effect of pimobendan or benazepril hydrochloride on survival times in dogs with congestive heart failure caused by naturally occurring myxomatous mitral valve disease: The QUEST study. J. Vet. Intern. Med..

[15] Pouchelon J.L., King J., Martignoni L., Chetboul V., Lugardon B., Rousselot J.F., Corlouer J.P., Bussadori C., Piette M.H., BENCH (BENazepril in Canine Heart disease) Study Group (1999). The Effect of benazepril on survival times and clinical signs of dogs with congestive heart failure: Results of a multicenter, prospective, randomized, double-blinded, placebo-controlled, long-term clinical trial. J. Vet. Cardiol..

[16] Sisson D.D. (1995). Acute and short-term hemodynamic, echocardiographic, and clinical effects of enalapril maleate in dogs with naturally acquired heart failure: Results of the Invasive Multicenter PROspective Veterinary Evaluation of Enalapril study: The IMPROVE Study Group. J. Vet. Intern. Med..

[17] Besche B., Blondel T., Guillot E., Garelli-Paar C., Oyama M.A. (2020). Efficacy of oral torasemide in dogs with degenerative mitral valve disease and new onset congestive heart failure: The CARPODIEM study. J. Vet. Intern. Med..

[18] Wess G., Kresken J.G., Wendt R., Gaugele J., Killich M., Keller L., Simak J., Holler P., Bauer A., Küchenhof H. (2020). Efficacy of adding ramipril (VAsotop) to the combination of furosemide (Lasix) and pimobendan (VEtmedin) in dogs with mitral valve degeneration: The VALVE trial. J. Vet. Intern. Med..

[19] Coffman M., Guillot E., Blondel T., Garelli-Paar C., Feng S., Heartsill S., Atkins C.E. (2021). Clinical efficacy of a benazepril and spironolactone combination in dogs with congestive heart failure due to myxomatous mitral valve disease: The BEnazepril Spironolactone STudy (BESST). J. Vet. Intern. Med..

[20] Peddle G.D., Singletary G.E., Reynolds C.A., Trafny D.J., MacHen M.C., Oyama M.A. (2012). Effect of torsemide and furosemide on clinical, laboratory, radiographic and quality of life variables in dogs with heart failure secondary to mitral valve disease. J. Vet. Cardiol..

[21] Pouchelon J.L., King J., Martignoni L., Chetboul V., Lugardon B., Rousselot J.F., Corlouer J.P., Bussadori C., Piette M.H., BENCH (BENazepril in Canine Heart disease) Study Group (2004). Long-term tolerability of benazepril in dogs with congestive heart failure. J. Vet. Cardiol..

[22] Ettinger S.J., Benitz A.M., Ericsson G.F., Cifelli S., Jernigan A.D., Longhofer S.L., Trimboli W., Hanson P.D. (1998). Effects of enalapril maleate on survival of dogs with naturally acquired heart failure. The Long-Term Investigation of Veterinary Enalapril (LIVE) Study Group. J. Am. Vet. Med. Assoc..

[23] Allen J.W., Phipps K.L., Llamas A.A., Barrett K.A. (2021). Left atrial decompression as a palliative minimally invasive treatment for congestive heart failure caused by myxomatous mitral valve disease in dogs: 17 cases (2018–2019). J. Am. Vet. Med. Assoc..

[24] Atkins C., Bonagura J., Ettinger S., Fox P., Gordon S., Häggström J., Hamlin R., Keene B., Luis-Fuentes V., Stepien R. (2009). Guidelines for the diagnosis and treatment of canine chronic valvular heart disease. J. Vet. Intern. Med..

[25] van Staveren M.D.B., Muis E., Szatmári V. (2023). Self-reported utilization of international guidelines for staging dogs with myxomatous mitral valve degeneration: A survey among veterinary practitioners. Vet. Sci..

[26] van Vuuren D., Vlaanderen M., Pomp M., Geelen J. SEO Arbeidsmarkt Dierenartsen. www.seo.nl.

[27] Buchanan J.W., Bücheler J. (1995). Vertebral scale system to measure canine heart size in radiographs. J. Am. Vet. Med. Assoc..

[28] Malcolm E.L., Visser L.C., Philips K.L., Johnson L.R. (2018). Diagnostic value of vertebral left atrial size as determined from thoracic radiographs for assessment of left atrial size in dogs with myxomatous mitral valve disease. J. Am. Vet. Med. Assoc..

[29] Wesselowski S., Gordon S.G., Meddaugh N., Saunders A.B., Häggström J., Cusack K., Janacek B.W., Matthews D.J. (2022). Prediction of clinically important acquired cardiac disease without an echocardiogram in large breed dogs using a combination of clinical, radiographic and electrocardiographic variables. J. Vet. Cardiol..

[30] Wilshaw J., Rosenthal S.L., Wess G., Dickson D., Bevilacqua L., Dutton E., Deinert M., Abrantes R., Schneider I., Oyama M.A. (2021). Accuracy of history, physical examination, cardiac biomarkers, and biochemical variables in identifying dogs with stage B2 degenerative mitral valve disease. J. Vet. Intern. Med..

[31] Rasmussen C.E., Falk T., Zois N.E., Moesgaard S.G., Häggström J., Pedersen H.D., Åblad B., Nilsen H.Y., Olsen L.H. (2012). Heart rate, heart rate variability, and arrhythmias in dogs with myxomatous mitral valve disease. J. Vet. Intern. Med..

[32] Baisan R.A., Vulpe V., Ohad D.G. (2021). Short-term heart rate variability in healthy dogs and dogs in various stages of degenerative mitral valve disease evaluated before pharmacotherapy. Vet. J..

[33] Häggström J., Hamlin R.L., Hansson K., Kvart C. (1996). Heart rate variability in relation to severity of mitral regurgitation in Cavalier King Charles spaniels. J. Small Anim. Pract..

[34] Ineson D.L., Freeman L.M., Rush J.E. (2019). Clinical and laboratory findings and survival time associated with cardiac cachexia in dogs with congestive heart failure. J. Vet. Intern. Med..

[35] Häggström J., Kvart C., Hansson K. (1995). Heart sounds and murmurs: Changes related to severity of chronic valvular disease in the Cavalier King Charles spaniel. J. Vet. Intern. Med..

[36] Duler L., Visser L.C., Jackson K.N., Phillips K.L., Pollard R.E., Wanamaker M.W. (2021). Evaluation of radiographic predictors of left heart enlargement in dogs with known or suspected cardiovascular disease. Vet. Radiol. Ultrasound.

[37] Ross E.S., Visser L.C., Sbardellati N., Potter B.M., Ohlendorf A., Scansen B.A. (2023). Utility of vertebral left atrial size and vertebral heart size to aid detection of congestive heart failure in dogs with respiratory signs. J. Vet. Intern. Med..

[38] Lofstedt J. (2003). Confidence and competence of recent veterinary graduates—Is there a problem?. Can. Vet. J..

[39] Bode E.F., Mederska E., Hodgkiss-Geere H., Radford A.D., Singleton D.A. (2022). Analysis of canine cardiovascular therapeutic agent prescriptions using electronic health records in primary care veterinary practices in the United Kingdom. J. Vet. Cardiol..

[40] Klein S., Nolte I., Rumstedt K., Sehn M., Raue J.F., Weiner F., Treese J.S., Beyerbach M., Bach J.-P. (2021). The effect of treatment with pimobendan in dogs with preclinical mitral valve disease—A placebo-controlled double-blinded crossover study. BMC Vet. Res..

[41] Oyama M.A., Scansen B.A., Boswood A., Goldfeder G., Rosenthal S., Cober R., LaFauci K., Friese R.C., Gomes M., Chang Y.R. (2023). Effect of a specially formulated diet on progression of heart enlargement in dogs with subclinical degenerative mitral valve disease. J. Vet. Intern. Med..

[42] Wesselowski S., Blake A.B., Gordon S.G., Suchodolski J.S., Steiner J.M. (2022). Whole blood and plasma taurine reference intervals in adult Cavalier King Charles Spaniels and correlations between taurine concentration, diet and mitral valve disease. J. Am. Vet. Med. Assoc..

[43] Chetboul V., Lefebvre H.P., Sampedrano C.C., Gouni V., Saponaro V., Serres F., Concordat D., Nicolle A.P., Pouchelon J. (2007). Comparative adverse cardiac effects of pimobendan and benazepril monotherapy in dogs with mild degenerative mitral valve disease: A prospective, controlled, blinded, and randomized study. J. Vet. Intern. Med..

[44] Franchini A., Borgarelli M., Abbott J.A., Menciotti G., Crosara S., Häggström J., Lahmers S., Rosenthal S., Tyrrell W. (2022). The Longitudinal Outcome Of canine (K9) myxomatous Mitral valve disease (LOOK-Mitral) registry: Baseline treatment characteristics. J. Vet. Cardiol..

[45] Donati P., Tarducci A., Zanatta R., Verdier N., Belerenian G., Cordero I., Villalta C., Franco J., Tarragona L. (2022). Angiotensin-converting enzyme inhibitors in preclinical myxomatous mitral valve disease in dogs: Systematic review and meta-analysis. J. Small Anim. Pract..

[46] Hammond H.H., Ames M.K., Domenig O., Scansen B.A., Tsang Yang N., Wilson M.D., Sunshine E., Brunk K., Masters A. (2023). The classical and alternative circulating renin-angiotensin system in normal dogs and dogs with stage B1 and B2 myxomatous mitral valve disease. J. Vet. Intern. Med..

[47] Häggström J., Hansson K., Karlberg B.E., Kvart C., Madej A., Olsson K. (1996). Effects of long-term treatment with enalapril or hydralazine on the renin-angiotensin-aldosterone system and fluid balance in dogs with naturally acquired mitral valve regurgitation. Am. J. Vet. Res..

[48] Ames M.K., Atkins C.E., Pitt B. (2019). The renin-angiotensin-aldosterone system and its suppression. J. Vet. Intern. Med..

[49] Kittleson M.D., Bonagura J.D. (2010). Letter to the editor. J. Vet. Intern. Med..

[50] Ames M.K., Atkins C.E., Eriksson A., Hess A.M. (2017). Aldosterone breakthrough in dogs with naturally occurring myxomatous mitral valve disease. J. Vet. Cardiol..

[51] Uechi M., Matsuoka M., Kuwajima E., Kaneko T., Yamashita K., Fukushima U., Ishikawa Y. (2003). The effects of the loop diuretics furosemide and torasemide on diuresis in dogs and cats. J. Vet. Med. Sci..

[52] Knauf H., Mutschler E. (1998). Clinical pharmacokinetics and pharmacodynamics of torasemide. Clin. Pharma.

[53] MSD Manual https://www.msdvetmanual.com/pharmacology/systemic-pharmacotherapeutics-of-the-cardiovascular-system/diuretics-for-use-in-animals.

[54] Wess G., Glaus T. (2021). Response to letter to the editor regarding “Efficacy of adding ramipril (VAsotop) to the combination of furosemide (Lasix) and pimobendan (VEtmedin) in dogs with mitral valve degeneration: The VALVE trial”. J. Vet. Intern. Med..

[55] Chetboul V., Pouchelon J.-L., Menard J., Blanc J., Desquilbet L., Petit A., Rougier S., Lucats L., Woehrle F. (2017). Short-term efficacy and safety of torasemide and furosemide in 366 dogs with degenerative mitral valve disease: The TEST Study. J. Vet. Intern. Med..

[56] Griffiths L.G., Orton C.E., Boon J.A. (2004). Evaluation of techniques and outcomes of mitral valve repair in dogs. J. Am. Vet. Med. Assoc..

[57] Uechi M., Mizukoshi T., Mizuno T., Mizuno M., Harada K., Ebisawa T., Takeuchi J., Sawada T., Uchida S., Shinoda A. (2012). Mitral valve repair under cardiopulmonary bypass in small-breed dogs: 48 cases (2006–2009). J. Am. Vet. Med. Assoc..

[58] Matsuura K., Yoshida T., Yamada S., Aboshi Y., Yotsuida H., Yaginuma Y., Hasegawa M. (2022). The outcome of surgical mitral valve repair with loop-in-loop technique in dogs with different stage myxomatous mitral valve disease. J. Vet. Cardiol..

[59] Bristow P., Markovic L.E. (2023). Mitral valve repair—The development and rise of options in the veterinary world. Vet. Clin. N. Am. Small Anim. Pract..

[60] Liu B., Leach S.B., Pan W., Zheng F., Jia L., Zhou X., Li J. (2020). Preliminary outcome of a novel edge-to-edge closure device to manage mitral regurgitation in dogs. Front. Vet. Sci..

